# Methane and Inflammation - A Review (*Fight Fire with Fire*)

**DOI:** 10.1186/s40635-019-0278-6

**Published:** 2019-12-05

**Authors:** Marietta Zita Poles, László Juhász, Mihály Boros

**Affiliations:** Institute of Surgical Research, University of Szeged, Pulz u. 1., Szeged, H-6724 Hungary

**Keywords:** Methanogenesis, Exogenous methane, Ischemia/reperfusion, Sepsis, Bioactivity

## Abstract

Mammalian methanogenesis is regarded as an indicator of carbohydrate fermentation by anaerobic gastrointestinal flora. Once generated by microbes or released by a non-bacterial process, methane is generally considered to be biologically inactive. However, recent studies have provided evidence for methane bioactivity in various in vivo settings. The administration of methane either in gas form or solutions has been shown to have anti-inflammatory and neuroprotective effects in an array of experimental conditions, such as ischemia/reperfusion, endotoxemia and sepsis. It has also been demonstrated that exogenous methane influences the key regulatory mechanisms and cellular signalling pathways involved in oxidative and nitrosative stress responses. This review offers an insight into the latest findings on the multi-faceted organ protective activity of exogenous methane treatments with special emphasis on its versatile effects demonstrated in sepsis models.

## Background

The human body uses and produces several gases. Nitric oxide (NO), carbon monoxide (CO) and hydrogen sulphide (H_2_S)—once considered to be toxic air pollutants—play a vital biochemical modulator role in living tissues. These small, volatile, available and biologically effective molecules are classified as ‘gasotransmitters’, which means that they take part in cellular communications. Methane (CH_4_) is also part of the gaseous environment which maintains the aerobic metabolism within the living system. If we discuss the available literature data on the generation and biological effects of CH_4_, the current evidence does not fully support the gasotransmitter concept, but it does support the notion that CH_4_ is bioactive. Several clinical studies have demonstrated that endogenous CH_4_ can modulate the signalling mechanisms of the enteric nervous system; in addition, exogenous CH_4_ has been proved to protect against organ damage in numerous experimental models associated with inflammation and/or ischemia/reperfusion (I/R) syndromes [5]. We briefly summarise the available data on the relationship between inflammatory activation and CH_4_ administrations with special emphasis on the possible mechanism of action. Papers that directly monitored sepsis- or endotoxin-linked organ dysfunction were then considered to illustrate the relationship between CH_4_ treatments and the effect on sepsis-related end organ dysfunction (Table 1).

**Table 1:** Summary of in vivo studies using CH_4_ that also monitored sepsis/LPS/surgery-induced organ dysfunction and other parameters of tissue damage

Reference	Experimental model/CH_4_ administration route	Target organ	Reported effects/main findings
Zhang X et al. [56]	Mouse + LPS Rat + *E. coli* Mouse + DSS MRS (16 ml/kg ip) pre-treatment	Colon Immune organs	Suppressed activation of NF-κB /MAPKs Increased survival Enhancement of IL-10 release
Sun A et al. [38]	Rat + LPS MRS (2 ml/kg and 20 ml/kg) pre-treatments	Lung	Reduction of acute lung injury Prolonged survival
Li Z et al. [23]	Mouse + CLP MRS (10 ml/kg ip) post-treatment	Liver	Reduction of sepsis-induced acute liver injury
Jia Y et al. [18]	Mouse + CLP MRS (10 ml/kg ip) post-treatment	Kidney	Reduction of sepsis-induced acute kidney injury
Li Z et al. [22]	Mouse + CLP MRS post-treatment	Lung Intestines	Inhibition of NOD-like receptor protein 3-mediated pyroptosis in vivo and in vitro
Bari G et al. [2]	Pig + ECC Inhalation of 2.5% v/v CH_4_ – normoxic air	Kidney	Higher renal blood flow during extracorporeal circulation
Zhang D et al. [58]	Mouse + abdominal surgery MRS (16 ml/kg ip) post-treatment	Brain	Reduction of postoperative cognitive dysfunction and microglial activation

*CLP*, cecal ligation and puncture; *DSS*, dextran sodium sulfate; *ECC*, extracorporeal circulation; *IL-10*, interleukin 10; *LPS*, lipopolysaccharide; *MAPKs*, mitogen-activated protein kinase; *MRS*, methane-rich saline; *NF-κB*, nuclear factor-κB

### CH_4_: a brief overview

CH_4_ is an intrinsically non-toxic, combustible gas which forms explosive mixtures with air at concentrations between 5% (lower explosive limit) and 15% (upper explosive limit) at room temperature. In humans, large amounts of CH_4_ can be produced by carbohydrate fermentation in the gastrointestinal (GI) tract through the metabolism of methanogenic microorganisms. The catalysing enzyme of this pathway is methyl coenzyme M reductase, while the microorganisms are obligate anaerobic Archae [9, 20, 21, 34, 49].

It should be added that relatively little is known about the in vivo roles of commensal methanogens in GI physiology because it is impossible to study or culture these microorganisms together with oxygen-requiring aerobic cells in conventional ways. The actual level of endogenous CH_4_ generation in the human body is still an open question. In general terms, about one-third of healthy adults emit gaseous CH_4_ identified with conventional breath testing, but a recent study using stable carbon isotopes and high-precision measurements provided evidence that exhaled CH_4_ levels were always above inhaled CH_4_ concentration [20]. Significant CH_4_ release was also demonstrated in previously non-CH_4_ producer volunteers after high ethanol intake [43]. Furthermore, in vitro and in vivo studies have revealed the possibility of non-microbial CH_4_ formation in mitochondria [29, 30] and eukaryotic cells, especially under hypoxic stress stimuli [14, 15, 44–46, 48]. Today, the sum of evidence suggests that a variable amount of excreted CH_4_ in the breath of mammals is possibly linked to non-archaeal processes [6, 42].

Another important issue is that due to its physico-chemical properties, intraluminal CH_4_ can traverse the GI mucosa and enter the splanchnic circulation freely. When reaching the lungs, the transported CH_4_ is partially released into the breath if the partial pressure is higher than that in the atmosphere, where it is normally about 1.8 parts per million volume (ppmv). Therefore, exhaled CH_4_ levels will change in relation to intestinal perfusion alterations, and variations in breath CH_4_ output may thus be related to the flow conditions of the mesenteric microcirculation as well [40].

### Endogenous CH_4_

Elevated breath CH_4_ concentrations have been traditionally linked to numerous GI health conditions, such as sugar malabsorption, small intestinal bacterial overgrowth or irritable bowel syndrome [7, 10, 13, 33, 41]. Further data suggest that endogenously produced CH_4_ can influence mammalian metabolism and thus energy homeostasis [5]. More importantly, higher CH_4_ concentrations in the GI tract can significantly slow transit time, while increasing the number of muscle contractions [17, 31]. It has been suggested that by slowing down the transit, the time for nutrient absorption is lengthened, which, together with boosted levels of methanogenic microorganisms in the intestines, could lead to an increased weight gain process and thus the development of obesity [3]. Indeed, a significantly higher ratio of H_2_-utilizing methanogen Archaea is associated with obesity [26, 27, 55].

### Effects of exogenous CH_4_

It should be noted that a shift in the energy balance may alter the inflammatory status as well. In this line, in a rat model of endurance exercise, treadmill running induced inflammatory activation, including leukocyte accumulation (evidenced by myeloperoxidase (MPO) activity, raised plasma levels of interleukins IL-1β, IL-6, IL-10 and tumour necrosis factor alpha (TNF-α)), while exogenous CH_4_ administration prolonged the running time and normalised the changes in blood lactate and glucose and the parameters of pro-inflammatory activation in the animals [52].

Indeed, it has been shown that exogenous CH_4_ efficiently influences many aspects of inflammatory pathologies [5, 6]. After the first study using a model of intestinal I/R, several series of analyses demonstrated that CH_4_-containing normoxic artificial air (2.2–2.5 v/v% CH_4_) has anti-inflammatory effects in I/R injuries with decreasing oxidative and nitrosative stress levels [4, 29, 32]. CH_4_ inhalation improved mitochondrial function by preserving the control levels of basal respiration and lowering cytochrome c activity [37]. Serosal microcirculation, the structure of small intestinal mucosa and the epithelial barrier function were also preserved by CH_4_ inhalation in a mesenteric I/R model [29]. In a recent study, where the nitrergic neuron numbers were characterised in adjacent intestinal segments before and after the occlusion of the superior mesenteric artery, exogenous CH_4_ inhalation significantly suppressed nitrotyrosine formation at all intestinal sites and protected the nitrergic neuron population [32]. Nitrotyrosine formation has been significantly suppressed by raising the CH_4_ input prior to stress induction in other animal models as well. In this line, exogenous CH_4_ lowered the malondialdehyde (MDA) and TNF-α levels, increased protein kinase B phosphorylation and hemoxygenase-1 (HO-1) expression, and significantly reduced neuronal deficit in a cerebral I/R model [57].

### CH_4_-enriched saline

CH_4_ can be administered in supersaturated methane-rich saline (MRS) solution as well (using 0.4 MPa pressure for 3 h). In this case, the concentration of CH_4_ does not drop significantly for 24 h [54] and remains relatively stable for over 4 weeks after production [8]. Using MRS, Ye et al. [54] first demonstrated that the plasma alanine aminotransferase (ALT) and aspartate aminotransferase (AST) levels were dose-dependently decreased after liver I/R. MRS also alleviated inflammation by reducing the elevated serum levels of interleukin-6 (IL-6), TNF-α, interleukin-1β (IL-1β) and interferon-γ (IFN-γ) and activated nuclear factor-κB (NF-κB) and mitogen-activated protein kinases (MAPK) both in concanavalin A–induced autoimmune hepatitis [16] and in carbon tetrachloride-induced liver injury [53]. In the latter case, MRS reduced the activation of chemokine ligand 1 (CXCL1), intercellular adhesion molecule-1 (ICAM-1) and MPO activity as well, while serum ALT and AST levels returned to control levels.

Recently, Wang et al. [47] demonstrated that MRS treatment is able to reduce the spleen weight, the disease activity index, the ulcer area and the histology score in acetic acid-treated mice with colitis. In this experimental series, MRS alleviated the inflammatory activation through reduced serum TNF-α and IL-6 and raised IL-10 levels. Oxidative stress with lowered tissue MDA levels and myeloperoxidase (MPO) activity and increased superoxide dismutase (SOD) and glutathione transferase (GSH) activity was reduced almost as effectively as after salazosulfapyridine treatment. Similar results have been presented in an acute pancreatitis model. The intraperitoneally applied MRS improved the tissue damage scores, exerted potent anti-apoptotic effects, inhibited the elevation of inflammatory cytokines (TNF-α, IL-6 and IFN-γ), elevated IL-10 levels, decreased tissue MPO activity and preserved SOD activity [51]. In a renal I/R model, MRS induced higher catalase (CAT) and SOD activity with diminished tissue MPO activity, MDA and 8-hydroxy-2′-deoxyguanosine (8-OHdG) levels, a lower rate of apoptosis was detected in the tissues, and suppressed blood urea nitrogen and creatinine, serum IL-6 and TNF-α levels were present. Again, this approach increased IL-10 concentration and decreased the number of F4/80^+^ macrophages in the renal tissue as well [28].

The anti-apoptotic effects of CH_4_ were first described in skin and liver I/R models [36, 54]. The authors have shown that MRS protects the transplanted skin flaps by reducing the leukocyte infiltration and lowering the apoptotic cell count, increases the expression of the anti-apoptotic B cell leukaemia/lymphoma-2 (Bcl-2) and attenuates the pro-apoptotic protein Bcl-2-associated X protein (Bax), the expression of pASK-1, p-JNK, and caspase-3 activity [36].

In a clinically relevant rat model, MRS treatment improved the cardiac function and prevented the formation of myocardial fibrosis in the long run 4 weeks after myocardial infarction. CH_4_ treatment reduced the level of myocardial necroenzymes, increased tissue SOD activity and GSH content, and lowered MPO activity, MDA and 8-OHdG levels, and xanthine oxidoreductase (XOR) expression. Again, the treatment decreased the number of apoptotic cells in addition to diminishing the expression of caspase-3 and caspase-9, and the Bax/Bcl-2 ratio [8].

There is also a growing amount of data on the neuroprotective properties of CH_4_. In a CO toxicity model, Fan et al. [12] demonstrated that MRS was protective against learning deficit, and Shen et al. [35] showed that MRS treatment raised the number of Nissl-stained cells and the potency to restore the CA1 region and cortex. Both research teams showed that MRS decreased tissue MDA, 3-nitrotyrosine and 8-OHdG levels, and plasma TNF-α and IL-1β levels, while increasing plasma IL-6 content and tissue SOD activity [12, 35]. MRS treatment reversed the changes in the thickness of the inner nuclear layer and the inner plexiform layer after diabetic retinopathy [50] and suppressed the reduction of retinal thickness after I/R [24]. In addition, MRS inhibited ganglion cell loss [24, 44–46, 50], improved the blood-retina barrier function [50], reduced visual dysfunction [24, 44–46], and lowered TNF-α- and IL-1β-positive cell numbers, and expression of VEGF and GFAP [50]. MRS decreased the overexpression of a mitochondrial biogenesis marker (PGC1-α) and restored citrate synthase activity, thus boosting ATP levels [44–46]. It also enhanced the upregulation of Bcl-2 and reduced the upregulation of Bax, caspase-3 and caspase-9, and cut oxidative stress levels by significantly suppressing 8-OHdG, 4-hydroxy-2-nonenal (4-HNE) and MDA levels, thus increasing SOD, CAT and glutathione peroxidase (GPx) activity [24]. In a spinal cord injury model, MRS treatment improved the Basso, Beattie and Bresnahan score over time, lowered microglial activation and inflammatory cell infiltration, raised tissue SOD activity and reduced MDA, TNF-α, IL-1β and IL-6 levels [44–46]. In a very similar spinal cord I/R study, MRS improved neurological damage, preserved the blood-spinal cord barrier, reduced oedema formation and leukocyte infiltration, and boosted SOD, CAT activity and GSH levels, while decreasing MDA, 8-OHdG and 3-nitrotyrosine levels. It also lowered the apoptotic cell number, caspase-3 and caspase-9 levels, and cytochrome c translocation into the cytoplasm, the mRNA and content of TNF-α, IL-1β, CXCL1 and ICAM-1 and the metalloproteinase MMP-9, while increasing the expression of the tight junction proteins claudin-5, occludin and ZO-1 [44–46]. The suppressed microglia and astrocyte activation, together with lowered CD3-positive T cell infiltration, was demonstrated in an arthritis model as well [59].

### Possible mechanisms of action

Due to its apolar properties, CH_4_ can be dissolved in cell membranes; it may thus be able to influence the physiochemical condition of the phospholipid bilayer [11]. Similar to halothane, which can influence the G-protein-mediated signalling pathways, CH_4_ is able to affect the function of transmembrane proteins, enzymes and ion channels [8]. Indeed, several studies have demonstrated the modulator effect of CH_4_ on cell-cell junctions and plasma membrane integrity under oxido-reductive stress conditions. Nevertheless, it has also been shown that CH_4_ can influence XOR activity and the isoform ratio of XOR; this facilitates its conversion into the xanthine dehydrogenase isoform, which then produces less reactive oxygen species (ROS) [4, 32]. XOR is not a transmembrane protein; thus, other possibilities, such as changing the hydrophobic environment around the FAD site, should be taken into account to understand the mechanism of CH_4_ action.

Interestingly, CH_4_ accumulation may directly influence intracellular signalling reactions, leading to anti-inflammatory responses via master switches, such as nuclear factor erythroid 2–related factor 2 (Nrf2)/Kelch-like ECH-associated protein 1 (Keap1) or NF-κB [56]. CH_4_ seems to have effects on the phosphoinositide 3-kinase (PI3K) pathway by facilitating the activation of Akt protein, thus increasing the expression of the HO-1 enzyme and promoting its anti-oxidative effects [57]. Moreover, also via the PI3K/Akt pathway, CH_4_ is able to influence the expression of the anti-inflammatory cytokine IL-10 through the activation of glycogen synthase-3β (GSK-3β). In a carbon tetrachloride–derived liver inflammation model, Yao et al. [53] blocked GSK-3β with wortmannin and pre-treated mice with anti-IL-10 antibody, which abolished the beneficial effects of MRS and raised the levels of phosphorylated NF-κB and MAPK proteins supporting the involvement of these pathways in the anti-inflammatory effects of CH_4_. In an acetic acid–induced ulcerative colitis model, MRS also proved its protective effects by blocking the expression of Toll-like receptor 4 (TLR4) and myeloid differentiation primary response 88 protein (MyD88) and attenuating the expression levels of p-NF-κB, p65, p-JNK, p-ERK and p-P38. On the other hand, CH_4_ could promote the expression of IL-10, Janus kinase 1 (JAK1), and signal transducer and activator of transcription 3 (STAT3), thus facilitating the anti-inflammatory response [47]. In another study, Wang et al. showed that MRS treatment facilitated the time-dependent nuclear translocation of Nrf2 in neurons, microglia and astrocytes after spinal cord injury. In addition, expression of the Nrf2 inhibitor Keap1 protein was inhibited and NF-κB translocation was blocked via this pathway [44–46].

### The effects of CH_4_ in endotoxemia and sepsis

**Fig. 1:**
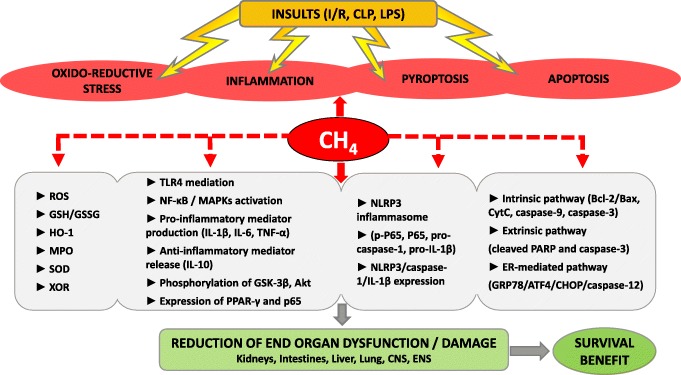
Documented CH_4_-mediated mechanisms in experimental models of sepsis, endotoxemia and systemic inflammation. ATF4, activating transcription factor 4; Bax. Bcl-2-associated X protein; Bcl-2, B cell lymphoma 2; CHOP, C/EBP homologous protein; CLP, cecal ligation and puncture; CNS, central nervous system; CytC, cytochrome C; ENS, enteric nervous system; ER, endoplasmic reticulum; GRP78, glucose-regulated protein 78; GSK-3β, glycogen synthase kinase 3 beta; GSH, glutathione; GSSG, glutathione disulphide; HO-1, heme oxygenase 1; IL-1β, interleukin 1 beta; IL-6, IL-10, interleukin 6 and interleukin 10, respectively; I/R, ischemia/reperfusion; LPS, lipopolysaccharide; MAPKs, mitogen-activated protein kinase; MPO, myeloperoxidase; NF-κB, nuclear factor-κB; NLRP3, NOD-like receptor protein 3; PARP, poly (ADP-ribose) polymerase; PPAR-γ, peroxisome proliferator-activated receptor gamma; ROS, reactive oxygen species; SOD, superoxide dismutase; TLR4, Toll-like receptor 4; TNF-α, tumour necrosis factor alpha; XOR, xanthine oxidoreductase

The effects of exogenous CH_4_ were tested in sepsis models as well, and it seems that CH_4_-mediated protection involves a triad of anti-inflammatory, anti-oxidative and anti-apoptotic actions (the mechanisms of CH_4_-mediated protection in experimental models of sepsis and endotoxemia are summarised in Fig. 1). Along with the ‘anti-effects’, anti-pyroptotic activity (a form of programmed cell death that occurs during infection) was recently also reported in lung and intestinal tissues in mice [22]. As the first steps of pyroptosis, NOD-like receptor protein 3 (NLRP3) inflammasome formation activates caspase-1, which in turn further drives inflammation via the cleavage of pro-IL-1β to IL-1β and pro-IL-18 to IL-18. Then an effector protein (Gasdermin D) is cleaved, and it forms pores within the membrane and induces pyroptosis [39].

Most importantly, MRS administration reduced cecal ligation and puncture (CLP)-induced endoplasmic reticulum stress in the kidney through the suppression of the GRP78/ATF4/CHOP/caspase-12-mediated apoptotic pathway [18]. Similar to the findings for MRS-treated ulcerative colitis [47] or autoimmune hepatitis [16] and in carbon tetrachloride-induced liver injury [53], CH_4_ also attenuated the lipopolysaccharide (LPS)-induced activation of MAPKs and NF-κB [56]. Moreover, CH_4_ enhanced GSK-3β activation, thus leading to an increased expression of the anti-inflammatory cytokine IL-10, similar to the findings for a carbon tetrachloride–derived liver inflammation model [53, 56].

All of these effects could collectively contribute to reduced cellular and tissue injury (i.e. in the lungs, intestines, kidneys and liver), amelioration of organ dysfunction (e.g. enzyme markers and histopathological scores) and increased survival [18, 22, 23, 38, 56]. Indeed, the 5-day and 7-day survival rates were boosted significantly after MRS therapies [22, 56].

Finally, it has been demonstrated that CH_4_ inhalation was able to reduce the systemic inflammatory response in a clinically relevant pig model of extracorporeal circulation (ECC). In this study, the inotropic demand was significantly lower, the renal XOR activity was reduced, the arterial flow was significantly higher, and the hour diuresis remained in the low normal range compared with the oliguria in the animals without CH_4_ treatment [2].

### Possible side effects of CH_4_

CH_4_ is a simple asphyxiant, meaning that it will displace oxygen in the air when present at about 14% in a restricted place. In such cases, the respiratory dysfunction is not due to the chemical specificity of the gas, but to the decreased oxygen content. Apart from CH_4_-induced asphyxia, there is very little known about the side effects of CH_4_ administration or how it may impact endogenous bacterial and non-bacterial productions. In a case report, Jo et al. [19] reported on CH_4_-caused acute respiratory distress, but it remained unclear whether this was related to the reduced oxygen content or due to the direct gas effect within the lung tissue. Similarly, the harmful effects of inhaled CH_4_ could not be proven by the study of Manning et al. [25], where lower O_2_ and extremely high CO_2_ levels were present together with the higher concentration of CH_4_. Data on human cardiovascular effects are sparse, but in a case report with a 45 min CH_4_ exposure, the unconscious patient had spontaneous breathing with an arterial pH value of 7.26 and made a full recovery later [1].

## Conclusion

The review of the available literature argues in favour of CH_4_ as a bioactive, therapeutic gas: exogenous CH_4_ improves cellular/organ function and increases survival in experimental models of inflammation, I/R, sepsis and endotoxemia. Future investigations should provide additional evidence for the efficacy of CH_4_-based treatments in other types of infectious disease models, filling the missing gaps in the still not fully understood cellular signalling pathways and the mechanism of action of CH_4_.

## Data Availability

The datasets generated and/or analysed during the current study are available in PubMed.

## Abbreviations

8-OHdG: 8-hydroxy-2′-deoxyguanosine
ALT: Alanine aminotransferase
AST: Aspartate aminotransferase
Bax: Bcl-2-associated X protein
Bcl-2: B cell leukaemia/lymphoma-2 protein
CAT: Catalase
CH_4_: Methane
CLP: Cecal ligation and puncture
CO: Carbon monoxide
CXCL1: Chemokine ligand 1
ECC: Extracorporeal circulation
GI: Gastrointestinal
GSH: Glutathione transferase
GSK-3β: Glycogen synthase-3β
H_2_S: Hydrogen sulphide
HO-1: Hemoxygenase-1
I/R: Ischemia/reperfusion
ICAM-1: Intercellular adhesion molecule-1
IFN-γ: Interferon gamma
IL-10: Interleukin 10
IL-1β: Interleukin 1 beta
IL-6: Interleukin 6
Keap1: Kelch-like ECH-associated protein 1
LPS: Lipopolysaccharide
MAPK: Mitogen-activated protein kinase
MDA: Malondialdehyde
MPO: Myeloperoxidase
MRS: Methane-rich saline
NF-κB: Nuclear factor-κB
NLRP3: NOD-like receptor protein 3
NO: Nitric oxide
Nrf2: Nuclear factor erythroid 2–related factor 2
ppmv: Parts per million volume
SOD: Superoxide dismutase
TNF-α: Tumour necrosis factor alpha
XOR: Xanthine oxidoreductase
